# Reaching exercise for chronic paretic upper extremity after stroke using a novel rehabilitation robot with arm-weight support and concomitant electrical stimulation and vibration: before-and-after feasibility trial

**DOI:** 10.1186/s12938-020-00774-3

**Published:** 2020-05-06

**Authors:** Yumeko Amano, Tomokazu Noma, Seiji Etoh, Ryuji Miyata, Kentaro Kawamura, Megumi Shimodozono

**Affiliations:** 1 Department of Rehabilitation and Physical Medicine, Kagoshima University Graduate School of Medical and Dental Sciences, 8-35-1 Sakuragaoka, Kagoshima, 890-8520 Japan; 2 Kagoshima University Hospital Kirishima Rehabilitation Center, Kagoshima, Japan; 3 Present Address: Department of Rehabilitation, Faculty of Health Science, Nihon Fukushi University, Higashi-nukumi-cho 26-2, Handa, Aichi 475-0012 Japan

**Keywords:** Stroke, Rehabilitation, Robotics, Reaching, Hemiparesis, Electric stimulation, Vibration, Exercise therapy

## Abstract

**Background:**

Our group developed a rehabilitation robot to assist with repetitive, active reaching movement of a paretic upper extremity. The robot is equipped with a servo motor-controlled arm-weight support and works in conjunction with neuromuscular electrical stimulation and vibratory stimulation to facilitate agonist-muscle contraction. In this before-and-after pilot study, we assessed the feasibility of applying the robot to improve motor control and function of the hemiparetic upper extremity in patients who suffered chronic stroke.

**Methods:**

We enrolled 6 patients with chronic stroke and hemiparesis who, while sitting and without assistance, could reach 10 cm both sagitally and vertically (from a starting position located 10 cm forward from the patient’s navel level) with the affected upper extremity. The patients were assigned to receive reaching exercise intervention with the robot (Yaskawa Electric Co., Ltd. Fukuoka, Japan) for 2 weeks at 15 min/day in addition to regular occupational therapy for 40 min/day. Outcomes assessed before and after 2 weeks of intervention included the upper extremity component of the Fugl-Meyer Assessment (UE-FMA), the Action Research Arm Test (ARAT), and, during reaching movement, kinematic analysis.

**Results:**

None of the patients experienced adverse events. The mean score of UE-FMA increased from 44.8 [SD 14.4] to 48.0 [SD 14.4] (*p* = 0.026, *r *= 0.91), and both the shoulder–elbow and wrist–hand scores increased after 2-week intervention. An increase was also observed in ARAT score, from mean 29.8 [SD 16.3] to 36.2 [SD 18.1] (*p* = 0.042, *r *= 0.83). Kinematic analysis during the reaching movement revealed a significant increase in active range of motion (AROM) at the elbow, and movement time tended to decrease. Furthermore, trajectory length for the wrist (“hand path”) and the acromion (“trunk compensatory movement”) showed a decreasing trend.

**Conclusions:**

This robot-assisted modality is feasible and our preliminary findings suggest it improved motor control and motor function of the hemiparetic upper extremity in patients with chronic stroke. Training with this robot might induce greater AROM for the elbow and decrease compensatory trunk movement, thus contributing to movement efficacy and efficiency.

*Trial registration* UMIN Clinical Trial Registry, as UMIN000018132, on June 30, 2015. https://upload.umin.ac.jp/cgi-open-bin/ctr/ctr_view.cgi?recptno=R000020398

## Background

Stroke is a leading cause of death and disability. In 2017, the number of patients treated for stroke in Japan was 1,115,000, with 109,844 deaths [1, 2]. Many survivors of stroke require nursing care to some extent; in fact, patients with stroke account for the largest percentage of claims under the Japanese Long-term Care Insurance System [3]. In a previous review, about 90% of patients with stroke had hemiparesis on admission, and less than 15% of them experienced complete motor recovery [4]. In stroke rehabilitation, some principles are well accepted: high-intensity, task-specific, goal-setting, and multidisciplinary-team care are needed to be effective [5]. Among these principles, “task-specific” might be controversial, because some theories of motor control suggest that, on the contrary, motor learning improves, and acquires greater generalizability, when a training program offers variability [6, 7]. The appropriate approach probably depends on the aim of rehabilitation (which can be subject-dependent): for example, a reaching movement with the arm is frequently needed in activities of daily living.

Robotic rehabilitation is a novel intervention method, and several reviews have noted that it leads to improved muscle strength and motor control of the affected upper extremity [8, 9]. A recent Cochrane review suggests that electromechanical and robot-assisted arm training might improve arm function, muscle strength of the upper extremity, and even activity of daily living after stroke [10]. Robotic devices can enable patients to perform task-specific, high-intensity rehabilitation due to increased repetition or amount of training.

At the same time, neuromuscular electrical stimulation (NMES) is widely employed as a rehabilitation technique. According to a previous study, NMES is effective at improving motor control and motor function of affected arms of patients with acute stroke [11], and the NMES system was more efficient when applied with a high-voltage pulsed current [12]. Although few studies have investigated untriggered NMES for the hemiparetic upper limb, continuous electrical stimulation with robotic training improved active range of motion and motor control [13], and we employed the NMES system without triggered electromyography (EMG) [14]. Continuous stimulation with NMES has been considered to be effective in facilitating contraction of paretic muscles [14]. Furthermore, the latest meta-analysis showed that electrical stimulation was effective for arm function and activity regardless of the stimulation type (NMES, EMG triggered, or sensory) [15].

Functional vibratory stimulation (FVS) is known to produce a favorable effect on spasticity, motor control, and gait after stroke [16]. Regarding hemiparetic upper extremities, previous studies have shown that focal vibration applied to paretic muscles is effective at decreasing spasticity with an amplitude of 91 Hz [17], and that it probably improves motor control with an amplitude of 120 Hz, especially in terms of smoothness of movement [18]. For the lower extremity, a previous study revealed that focal vibration improved gait by promoting contraction of the target muscle [19]. Moreover, not only did it promote contraction of the agonist muscle, low amplitude vibratory stimulation (80 Hz) also facilitated focused motorcortical activation [20, 21]. In addition, tendon or muscle vibration produces a tonic vibration reflex through both spinal and supraspinal pathways via repetitive activation of Ia afferent fibers [22, 23]. It is possible to artificially elicit the illusion of movement by vibrating the tendons or the muscles through the skin [24]; the illusion is probably mediated by the activation of muscle spindles [25]. This phenomenon indicates that vibration induces a strong proprioceptive feedback. On the other hand, it has been reported that the vastus lateralis muscle demonstrates a shift toward more appropriate muscle timing when vibration is applied during stance phase and transition to stance of the gait cycle in patients with spinal cord injury [26]. This indicates that strong sensory feedback from quadriceps vibration caused increased muscle excitation [26]. Thus, the combination of muscle vibration with NMES might help to recruit Ia afferent fibers and increase muscle force production. This phenomenon has already been demonstrated in healthy people in the plantar flexors [27]. To the best of our knowledge, however, the use of a robotic device equipped with electrical stimulation and vibration has not been reported.

Considering these facts, our group undertook to develop a rehabilitation robot to assist with repetitive, active reaching movement of the paretic upper extremity; patent acquisitions [28–30] and product development were accomplished with a medical–engineering collaboration within Kagoshima University and collaboration between industry (Yaskawa Electric Co., Ltd., Fukuoka, Japan) and academia (Kagoshima university). The robot is equipped with a servo motor-controlled arm-weight support via a wire—the system is programmed to assist the patient’s paretic arm to move between two switches (sensors) located at various three-dimensional positions, which provide a variety of reaching tasks—and works in conjunction with NMES and vibratory stimulation to facilitate agonist-muscle contraction, because the combination might strengthen proprioceptive feedback and tonic vibration reflex. Indeed, this device was applicable and beneficial for a patient with incomplete spinal cord injury [31]. In the before-and-after pilot study reported here, we assessed the feasibility of our novel approach of applying the robot equipped with electrical stimulation and vibration to improve motor control and function of the hemiparetic upper extremity in patients who suffered chronic stroke.

## Results

All six patients fully accomplished the procedure, including assessments, before and after the intervention. No adverse events were observed during the study.

### Clinical measures

The changes in the UE-FMA and ARAT scores before and after 2 weeks intervention are shown in Table 1. UE-FMA and ARAT scores increased significantly. After the intervention, the mean UE-FMA score was significantly higher (*p* = 0.026, *r *= 0.91). Both the shoulder–elbow score (*p* = 0.041, *r *= 0.83) and the wrist–hand score (*p* = 0.039, *r *= 0.84) of UE-FMA were significantly higher. A significant increase was also observed in ARAT scores (*p* = 0.042, *r *= 0.83).

**Table 1:** Changes in clinical outcome measures

Patient	Pre-intervention				After 2 weeks intervention			
	UE-FMA scores	Shoulder–elbow FMA	Wrist–hand FMA	ARAT scores	UE-FMA scores	Shoulder–elbow FMA	Wrist–hand FMA	ARAT scores
1	58	37	21	42	62	39	23	52
2	34	28	6	25	36	29	7	25
3	52	34	18	44	56	35	21	51
4	49	31	18	43	51	31	20	51
5	55	31	24	22	58	34	24	30
6	21	18	3	3	25	20	5	8
Mean	44.8	29.8	15.0	29.8	48.0*	31.3*	16.7*	36.2*
SD	14.4	6.6	8.5	16.3	14.4	6.5	8.4	18.1

*UE-FMA* upper extremity component of the Fugl-Meyer Assessment, *ARAT* Action Research Arm Test, *SD* standard deviation

*p value of < 0.05, compared post 2-week intervention with pre-intervention using Wilcoxon signed-rank test

Changes in MAS scores were not statistically significant. The mean MAS score assessment did not change in terms of the wrist flexor muscles (from 0.5 [SE 0.2] to 0.5 [SE 0.3]; *p* > 0.999). It decreased slightly in the elbow flexor muscles (from 1.3 [SE 0.5] to 1.0 [SE 0.4]; *p* = 0.157) and in the flexor digitorum muscles (from 0.3 [SE 0.2] to 0.2 [SE 0.2]; *p* = 0.317).

### Kinematic analysis

All of the participants achieved task movements with the target button placed 10 cm or 20 cm from the start button in the sagittal and vertical directions. Only four of the six patients reached the target button when attempting to reach 30 cm from the start button. Three patients could reach the ipsilateral target and two patients could reach the contralateral target. Therefore, the movement time and the trajectory length were compared only among participants who achieved the task movement. However, AROM of the elbow and shapes of trajectories were measured and compared in all conditions, even if a patient could not successfully perform the tasks.

The AROM of the elbow for all targets tended to increase after the completion of the intervention, and it increased significantly for the target 30 cm from the start button (*p *= 0.028, *r *= 0.9), as well as in the contralateral workspace (*p *= 0.028, *r *= 0.9). On the other hand, AROM showed no significant change when the target button was in the ipsilateral workspace (*p *= 0.116, *r *= 0.64). Changes in AROM of elbow for the three above-mentioned conditions are reported in Table 2.

**Table 2:** Changes in AROM of the elbow

Pt.	Conditions for the task movement					
	30 cm		Ipsilateral		Contralateral	
	Pre	Post	Pre	Post	Pre	Post
1	65.5	67.3	79.1	77.9	76.2	76.5
2	45.2	56.8	77.3	90.8	45.1	62.4
3	45.2	48	60.7	58.2	57.9	61.5
4	40.8	53.4	45.5	64.1	33.8	43.7
5	48.4	78.7	72.8	82.6	73	75.1
6	32.2	60.1	38.5	56.4	30.8	43.4
Mean	46.2	60.7*	62.3	71.7	52.8	60.4*
SD	11	10.9	17.1	14.1	19.4	14.5

*Pt.* patient, *Pre* pre-intervention, *Post* post 2-week intervention, *SD* standard deviation. All values are presented with degree of angles

*p value of < 0.05, comparing post 2-week intervention with pre-intervention

Movement time while reaching showed no significant changes. However, mean values of movement time generally became shorter in all conditions, especially among participants who completed the task.

Table 3 shows changes in trajectory lengths for reaching to the target 20 cm from the start button before and after intervention. All participants completed this task successfully. Trajectory length tended to decrease with both wrist and shoulder in all five conditions, although none of the changes was statistically significant.

**Table 3:** Changes in trajectory length

Pt.	Pre-intervention		After 2 weeks intervention	
	Shoulder	Wrist	Shoulder	Wrist
1	16	93	16.5	78.1
2	31.4	77.8	14.8	70.2
3	18.2	57.3	11.3	44.9
4	17.5	63.5	13.7	61.5
5	34.2	87.6	33	84.3
6	25.4	73.7	25.1	87.5
Mean	23.8	75.5	19.1	71.1
SD	7.8	13.7	8.3	15.9

Condition for the task movement: the target button 20 cm away from the start button in the sagittal and vertical directions. *Pt.* patient, *SD* standard deviation

All values are presented with centimeter. Changes were not statistically significant comparing post-2-week intervention with pre-intervention

Trajectory shape for the wrist tended to be close to a straight line from the start button to the target button, or raised smoothly after 2 weeks intervention, which was consistent with the tendency towards decreasing trajectory length.

**Fig. 1:**
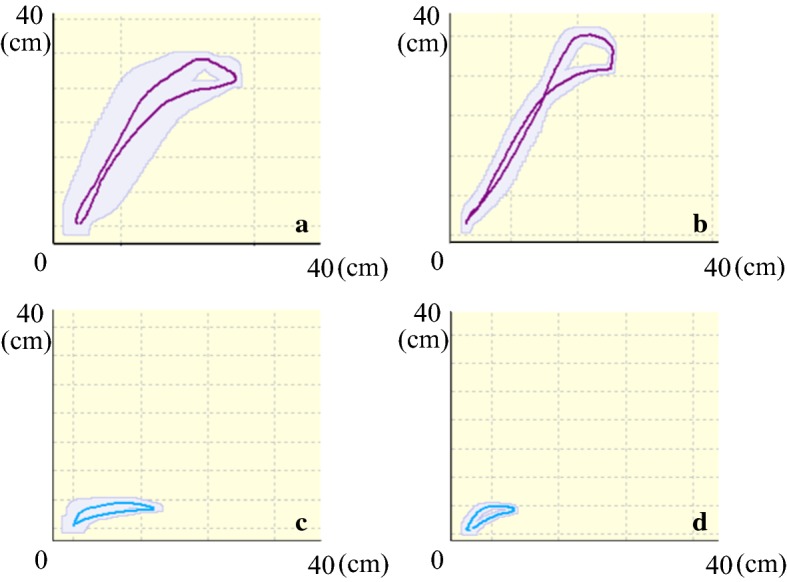
Trajectories of patient 2. Condition for the task movement: the target button 30 cm away from the start button in the sagittal and vertical directions. The vertical axis expresses height and the horizontal axis expresses forward length; each box is a square with sides 40 cm long. The start button is located at the bottom left, and the target button is located towards the upper right. The trajectory is shown as a standardized thick line. Compared with the task before intervention (**a**), the trajectory of the wrist after intervention raised smoothly (**b**), although it was slightly above the target. At the same time, the trajectory of the shoulder after intervention (**d**) was shortened in the direction of the horizontal axis compared with the shape before intervention (**c**)

Figure 1 shows trajectories of patient 2 as an example. The trajectory (standardized thick line) of the wrist after intervention raised smoothly to the target, although it was slightly above the target (Fig. 1b). Meanwhile, the trajectory of the shoulder shortened in the direction of the horizontal axis (Fig. 1d). The SD of trajectories (areas around the standardized lines, displayed in Fig. 1b, d) in wrist and shoulder decreased after intervention.

**Fig. 2:**
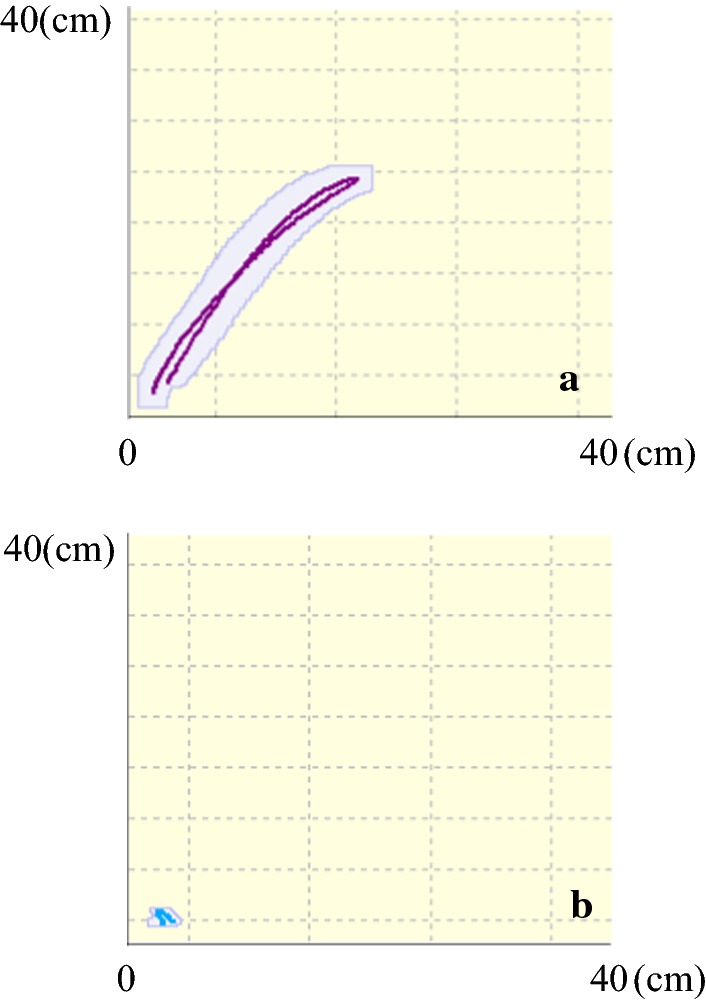
Trajectories for the non-paretic arm of patient 2. Condition for the task movement: the target button 30 cm away from the start button in the sagittal and vertical directions. The wrist trajectory (**a**) showed a nearly straight line from the start button to the target button, and the shoulder (**b**) did not shift forward

Additionally, trajectories of reaching movements with non-paretic arms appeared to be considerably tight for both shoulder and wrist of all patients. The result for the non-paretic arm of patient 2 is presented in Fig. 2.

Furthermore, we considered seven aspects of kinematic analysis, including some that are subjective, to further understand individual patient trajectories during the reaching condition in which the patient could reach the maximum distance both before and after the intervention; four out of the six patients (patients 1 to 4) reached the target button 30 cm away from the start button in the sagittal and vertical directions, and two out of the six patients (patients 5 and 6) reached the target button 20 cm away in the same directions (Table 4).

**Table 4:** Insights of changes in trajectories after 2 weeks intervention

Pt.	Wrist			Shoulder			Change of movement pattern
	Movement time	Trajectory length	Deviation	Trajectory length	Deciation	Forward displacement	
1	−	−	−	+	Unclear	+	
2	−	+	−	−	−	−	
3	−	−	Unclear	−	Unclear	−	+
4	−	−	−	−	−	−	+
5	+	−	Unclear	−	Unclear	−	+
6	−	++	Unclear	−	Unclear	±	

Conditions for the task movement were that the individual patient (Pt.) could reach the maximum distance both before and after the 2 weeks intervention; Pt. 1 to 4 reached the target 30 cm away from the start in the sagittal and vertical directions, and patients 5 and 6 reached the target 20 cm away in the same directions. +, increase; −, decrease; these simply mean the changes of values, not significant changes in the mean. In the column “Change of movement pattern” only, “+” means that the movement pattern of the wrist had changed. “Unclear” was only assessed by the shapes of the trajectories

**Fig. 3:**
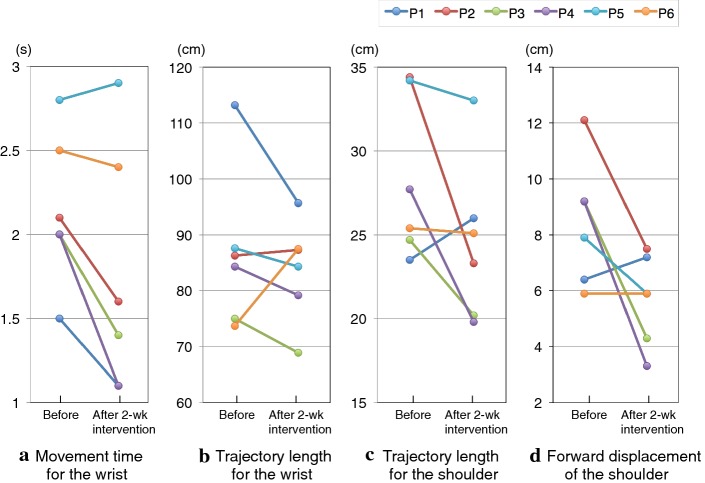
Changes in objective assessment for kinematic analysis before and after 2-week intervention for all six patients. Conditions for the task movement were that individual patient (Pt.) could reach the maximum distance both before and after the 2 weeks intervention; Pt. 1 to 4 reached the target 30 cm away from the start in the sagittal and vertical directions, and patients 5 and 6 reached the target 20 cm away in the same directions. **a** Movement time for the wrist, **b** trajectory length for the wrist, **c** trajectory length for the shoulder, and **d** forward displacement for the shoulder. P1 to P6 correspond to patients 1 to 6 in the tables, respectively

Changes in movement time of the wrist, trajectory length for the wrist and shoulder, and forward displacement of the shoulder are objective assessments (Fig. 3, Table 4). As shown in Fig. 3, the movement time tended to decrease (*p *= 0.058, *r* = 0.77). Although there was no significant change in trajectory length for the wrist (*p *= 0.345, *r* = 0.39), the length trended to decrease for the shoulder (*p *= 0.116, *r *= 0.64). In addition, forward displacement for the shoulder tended to decrease (*p *= 0.08, *r *= 0.72) (Table 4).

We also made three subjective assessments: deviations of trajectory for the wrist and shoulder, and changes in movement pattern revealed by the trajectory (Table 4). Movement pattern, which is presumed to indicate the movement strategy, apparently changed in three patients, although it is a subjective assessment. Patient 3 became able to raise her arm smoothly; patient 4 did likewise, and he might have gotten used to the reaching movement because the smooth shape after intervention implies that the time from pressing the target button to pulling his arm down had shortened.

## Discussion

This study revealed the feasibility of using a robotic device equipped with electrical stimulation and vibration for rehabilitation of patients with hemiparetic upper limb due to stroke. All six of the patients in this study completed a 2-week intervention without adverse events and showed improved motor control and motor function.

We found that the mean UE-FMA scores increased by 3.2 points. This is the same as the minimal detectable change [32]. Hence, a 2-week intervention might contribute to improvement of motor control of the affected upper-limb. Although changes in scores did not exceed minimal detectable change, we should take account of the current intervention period (2 weeks) when comparing to previous studies. In a previous systematic review and meta-analysis by Veerbeek et al., which investigated the effects of robot-assisted therapy for the paretic upper limb, the mean UE-FMA in the robot-assisted therapy groups was 2.23 points better than in the control groups [8]. On the other hand, the most recent multicenter randomized controlled trial (RCT) reported a mean improvement of 2.54 (0.07 to 5.06) points over usual care at 6 months [33]. In terms of the patient with chronic stroke, such RCTs showed that the robotic rehabilitation group had mean increases in the UE-FMA: 3.25 points after robotic rehabilitation at least 45 min three times a week for 8 weeks (total 24 sessions) [34]; approximately 4 points after 60 min three times a week for 6 weeks (total 18 sessions) [35]; and 3.87 points after high-intensity, repetitive, task-oriented movements (1024 per session on average) for 12 weeks (total 36 sessions) [36]. The current shoulder–elbow scores, including coordination, increased by 1.5 points and the wrist–hand scores increased by 1.7 points. Therefore, for both proximal and distal upper extremities, the intervention in the current study appears to have been effective.

Mean ARAT score changed by 6.4 points, which exceeds the minimal clinically important difference (5.7 points) for patients with chronic stroke [37]. Few studies of robotic rehabilitation have assessed ARAT. The latest RCT compared motor functions of paretic upper extremities among robotic training, intensive training, and usual care [33], but unlike FMA, no significant improvement compared with the controls in ARAT was revealed. However, if “instrumented ARAT” had been used in the previous RCT, a difference might have been detected in subtle arm alterations due to the more sensitive quantification of arm function [38]. Given that fact, our current results seem to be meaningful. In the present robot-assisted modality, the electrical stimulation, and the vibration are applied to the proximal region of the upper limb. However, improvement of shoulder–elbow (proximal) function might also affect stabilizing proximal function during manipulation of objects with regular occupational therapy or in activities of daily living. Although participants were only asked to repeat a reaching movement forward and upward in this study, it is considered that such repetitive task training was also effective to improve motor function in the paretic upper limb.

The reaching exercise with this robotic device—in addition to regular occupational therapy—appears to be effective for patients with chronic stroke. The strongest aspect of the device seems to be supporting the affected arm against gravity. In previous studies, gravity-supported exercise for patients with chronic stroke with hemiparesis provided functional recovery of upper extremities measured by the FMA [39, 40]. In addition, in the current study an adjusting system controlled by a servo motor, which could adjust the amount of arm weight relieved depending on the ability of patient, might allow the patient to more comfortably perform a greater number of repetitive reaching exercises. However, the amount of arm-weight support was not predefined in the present machine, nor was it obtained after calculating motor torque. This could be improved by determining the actual percentage of weight reduced in relation to the patient’s arm. This differs from patient to patient, so a relationship based on an individual’s weight could be used to better estimate the force acting to leverage the arm’s weight. The estimated amount of support could then be regulated according to the patient’s requirements. For instance, the approximate weight of the total arm is about 4.9% of total body weight [41], so a Japanese arm will weigh about 2.5 kg. In the current study, therefore, about half of arm weight was reduced because the force reduced by the robot was set between 1200 and 1500 g. However, we do not know whether this weight relief is optimal for rehabilitation of the paretic arm. Accordingly, the appropriate amount of support should be examined in the future for patients with hemiparesis, in the same way as it was examined by Coscia et al. with healthy subjects [42].

“Tonic vibration reflex increases with the initial muscle contraction and increases with vibration frequency up to 100–150 Hz but decreases beyond” [43]. Thus, in the current study, FVS was used with an amplitude of approximately 100 Hz and was applied to muscles of the affected side only while the patient was reaching for the target. FVS might therefore be able to decrease excessive spasticity and promote agonist-muscle contraction.

High-intensity and task-specific training are considered important for stroke rehabilitation [5, 44, 45]. Robot-assisted therapy contributes to high-intensity practice due either to an increase in number of repetitions [46] or to the robot being added to regular practice [9], or to both. The frequency of repetitions of reaching movements varied from patient to patient, ranging from 200 to 700 per 15-min session. Although the frequency was smaller than in previous studies [36, 47, 48], the increases in outcome measures that we observed suggest that our lower frequency of repetitions was not inferior to that in other studies. Thus, our reaching robot might enable a participant to experience an efficient and effective rehabilitation exercise in 2 weeks of intervention. In the same way, the duration of intervention per day could be kept short. However, the most appropriate intensity, as to the repetitive numbers and the training time, should be reconsidered in a future study.

As we describe and show in Table 4 and Fig. 3, certain aspects of kinematic analysis were defined in a previous review concerning robotic rehabilitation of the paretic upper limb and assessment of movement quality [49]. According to this review, “temporal efficiency”, the time required to perform the movement, is expected to decrease with recovery. The current results revealed a tendency towards reductions in movement time. Furthermore, the review noted that “efficient movement” implies the shortest possible trajectory. In the current study, trajectory length after intervention tended to be shorter than that before intervention.

The exact mechanism of motor improvement induced by robotic rehabilitation has not yet been generally understood. Kinematic analysis is the preferred method to quantify improvement in motor performance and to elucidate the mechanism of motor learning in robot-assisted rehabilitation [8]. While many indicators have been proposed for kinematic analysis for the hemiparetic upper limb after stroke, active range of motion (AROM), movement time, and trajectory length are frequently described, although specifics of their use differ slightly [50–53]. Previous studies in which paretic upper limb kinematics were assessed during a movement task have reported a tendency for AROM in the elbow joint to increase and movement time to decrease [50–53]. Furthermore, trunk compensation with paretic upper-limb movement was assessed in previous studies [52, 54], although a consistent conclusion has not been established. We obtained results that are similar in several ways to results of previous studies, as follows.

An increase in AROM of the elbow was considered to indicate improved movement efficacy, and a decrease in movement time suggests improved efficiency in the paretic upper limb [49].

The trajectory length traced by the wrist decreased in five out of six patients (Table 3). In previous studies, an indicator of trajectory was often used to express movement efficiency or accuracy [55]. In the current study, we considered a decreased wrist trajectory length to signify improvement of movement efficiency facilitated by a reduction of wasted effort. Only patient 6 showed an increase in trajectory length (see in Table 3), but we do not consider it a worsening of performance. We speculate that it is because the patient had more severe paresis (UE-FMA scored 21) than the other five patients, so the task performance was insufficient before the intervention. As this patient’s AROM increased after intervention (as seen in Table 2), it might result in a longer path length than before intervention.

A reduction in the trajectory length traced by the shoulder and the forward displacement of the shoulder (Fig. 3, Table 4) were considered as a decrease in trunk compensation during the reaching movement. Although statistically significant changes were not seen in the current results, patients 2, 3, and 4 showed a reduction in trunk compensation. Such reduction might indicate improvement in movement efficacy.

Limitations of this study are the small sample size and lack of a control group. The small number of subjects prevents us from generalizing our results to all patients with stroke and hemiplegia. An improvement in the patients has been reported here, but whether it was due to exercise with the robot, to the regular occupational therapies, or to the combination of both is not clear because there was no control group. Given the overall function of the paretic upper-extremity clinically—the improvement of proximal and distal function—the machine was relatively focused on improving proximal function. When planning the current feasibility study, therefore, we had thought that regular occupational therapy, which primarily focuses on enhancing distal function (i.e., hand dexterity), would need to be added to the robotic training. In addition, the time between stroke onset and enrollment in the study was quite long. Patient 2, with the longest elapsed time (about 12 years) did not show improvement in ARAT score (see in Table 1). However, a feasibility study could benefit by being conducted with a range of patient conditions. Furthermore, the effect of arm-weight support, NMES, and FVS on recovery of upper-limb function was not proven in this study, because we utilized a before-and-after design during only 2 weeks of intervention due to limited length of stay in the hospital, and the intervention included regular occupational therapy for 40 min per day. A future study, accordingly, needs to address the effect of the three components individually or in combination with a larger RCT design and with longer intervention and follow-up periods. In addition, it is not yet clear which indicator of kinematic analysis best suggests improvement in upper limb movement. However, the parameters we used have been employed widely in previous studies. Parameters related to speed and acceleration, such as the normalized jerk and number of velocity peaks that are often used as the standard approach for evaluating smoothness [56, 57], should be incorporated in kinematic analysis to allow further understanding of the mechanism of motor improvement induced by robot-assisted rehabilitation. A future study should recruit larger numbers of participants and define distinct indicators of kinematic analysis.

## Conclusion

Our robot-assisted modality is feasible and our results suggest that it might improve motor control and motor function of the hemiparetic upper extremity in patients who suffered chronic stroke. Training with this robot might enhance AROM of the elbow and decrease compensatory trunk movement, thus contributing to movement efficacy and efficiency.

## Methods

### Subjects

Subjects were recruited from patients admitted to the Kagoshima University Hospital Kirishima Rehabilitation Center, Japan, from June 30, 2015 to May 12, 2017. Inclusion criteria were as follows: (1) age between 20 and 80 years; (2) hemiparesis of the upper extremity with a diagnosis of first-time stroke (hemorrhage or infarction); (3) at least 24 weeks after onset of unilateral cerebral hemisphere stroke; (4) Brunnstrom recovery stage (BRS) of the upper extremity [58] ≥ 4; (5) ability to sit in a chair without assistance for 15 min; (6) ability to reach 10 cm both sagittally and vertically from the starting position with the affected upper extremity. Specifically, the starting position was set to be front of the patient, along with the sagittal direction between the patient’s navel and two buttons of the robot. In addition, the start button was set 10 cm away from the patient’s navel with the height at the navel, and the target button was set 10 cm farther from the start button in a sagittal line with the height at 10 cm above the start button. Exclusion criteria were as follows: (1) any medical condition for which electrical stimulation and vibration are contraindicated; (2) severe contractures, pain, or sensory disturbance of the affected upper extremity; (3) cognitive disorders that would interfere with understanding the procedure; (4) any other neurological or orthopedic diseases; (5) addition or dosage change—during the intervention—of any of the following prescription medicines that could influence upper limb function and performance: anti-spasticity drugs, dopaminergic drugs, antidopaminergic drugs, benzodiazepines and donepezil hydrochloride; (6) concurrently receiving botulinum injections (within 4 months), transcranial magnetic stimulations, or transcranial direct current stimulations.

Six patients with stroke (mean age, 58.2 years [SD 12.3]; range 38–66; 4 men and 2 women) met the inclusion criteria and were assigned to this study. All of the patients in this study had been diagnosed with cerebral infarction (2 subcortical, 2 basal ganglia, 1 capsula interna, 1 corona radiata). The mean time after stroke onset was 176 weeks (SD 208.5; range 38–593). Characteristics of participants are presented in Table 5.

**Table 5:** Participant characteristics

Patient	Age, years	Sex	Handedness	Diagnosis	Site of lesion	Side of hemiplegia	Time after stroke onset, weeks	BRS (upper limb/hand)
1	66	M	R	CI	Basal ganglia	R	54	5/5
2	73	M	R	CI	Subcortical	L	593	4/5
3	38	F	R	CI	Capsula interna	R	38	5/5
4	64	M	R	CI	Subcortical	R	107	5/5
5	53	F	R	CI	Basal ganglia	R	153	4/5
6	55	M	L	CI	Corona radiata	R	111	4/3

*BRS* Brunnstrom recovery stage; *M* male, *F* female, *R* right, *L* left, *CI* cerebral infarction

### Study design

This study utilized a before-and-after pilot design to examine feasibility and effectiveness of the reaching robot. Patients who met the inclusion criteria and did not fail the exclusion criteria received reaching exercise with the robot for 2 weeks at 15 min per day in addition to regular occupational therapy for 40 min per day.

### Intervention

**Fig. 4:**
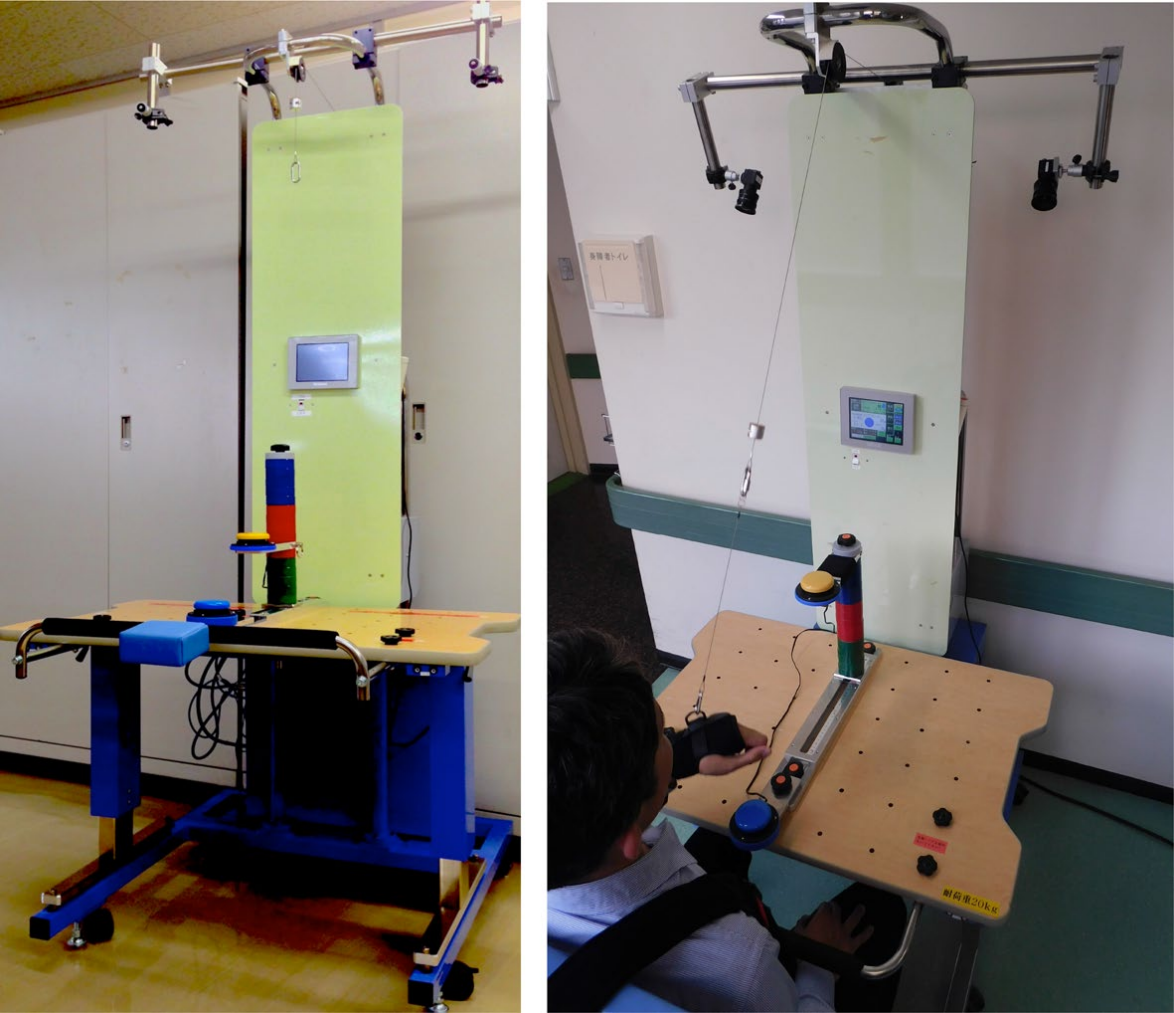
The reaching robot (Yaskawa Electric Co., Ltd. Fukuoka, Japan). The target button is yellow and the start button is blue

A reaching robot (Yaskawa Electric Co., Ltd. Fukuoka, Japan) with servo motor-controlled arm-weight support and concomitant electrical stimulation and vibration was employed in the intervention. This system (Fig. 4) has two video cameras on its upper frame for recording the patient’s movement. Features of this robot include (1) adjustment of the height, distance, and direction of the target button within reaching area, (2) adjustment of the amount of weight support for the arm (range 500–2500 g), and (3) facilitation with NMES and FVS to agonist muscles. The robot used in the current study is a prototype of a commercial version “Arm Rehabilitation Robot” (CoCoroe AR^2®^, Yaskawa Electric Co., Ltd., Fukuoka, Japan). However, the commercial version is not equipped with video cameras.

**Fig. 5:**
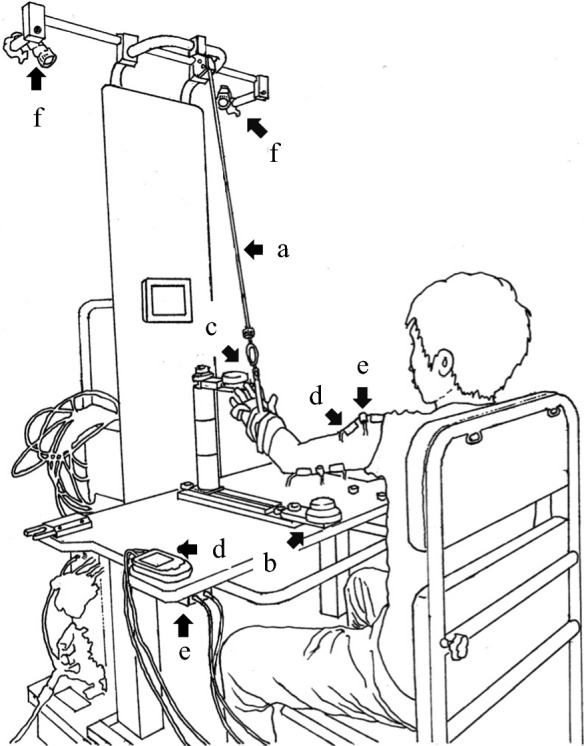
Setting for training with the robot. A wire (**a**) connecting the device to the forearm cuff adjusts the amount of arm-weight support. The patient repeats a reaching movement from the start button (**b**) to the target button (**c**), accompanied with the arm-weight support, electrical stimulation (**d**), and vibratory stimulation (**e**). Two video cameras (**f**) on the upper frame of the device record the reaching movement for kinematic analysis

Participants performed reaching exercises in the horizontal and vertical planes while seated in a chair with a backrest, with their hip joints and knee joints flexed at 90 degrees. The seat height was adjusted to the participant’s lower leg length. The table height was adjusted to the level of the participant’s navel, which is the same height as the start button. The target button was in the sagittal direction; the height and the distance of the target button were determined by the occupational therapist. The participant’s arm was supported by the forearm cuff and the wire connected to the servo motor-controlled arm-weight support system. The primary functional elements of this robot are illustrated in Fig. 5.

The amount of support (grams, g) was set for each participant by the occupational therapist in charge of the patient to allow a “comfortable” repetitive reaching exercise during 15 min. The amount of arm-weight support (i.e., extent of causing the arm to move upward and forward) could be adjusted “assist-as-needed” during reaching to the target button (forward path) and returning to the start button (backward path). As a result, the amount of weight support was approximately 1200 to 1500 g during reaching to the target button (forward path), and 700 to 900 g during returning to the start button (backward path). NMES was applied to the anterior part of the deltoid muscle and the triceps brachii muscle via a pair of surface electrodes, and FVS was applied with a vibrating motor (Fig. 5).

NMES (ITO ESPURGE, Ito Co., Ltd. Tokyo) was set to continuous mode, and the stimulation pulse was a symmetrical biphasic waveform with pulse width 250 μs and frequency 50 Hz. Intensity of the electrical current was adjusted to achieve slight contraction of the agonist muscle without visible movements of the limb or joint [14]. FVS (Vibrating motor: FM34F, Tokyo Parts Industrial Co., Ltd. Isesaki, Japan) was set to work only during the reaching movement (forward path; in other words, while the patient was extending the arm from start to target button); the vibrating motor would be switched on when the start button was pressed and switched off when the target button was pressed. Frequency of vibration was fixed to approximately 100 Hz. Participants were instructed to perform the reaching exercise at their preferred speed under the supervision of the occupational therapist. Patients received robotic training for 15 min and regular occupational therapy for 40 min per day for 2 weeks by the same occupational therapist in charge of each patient. Regular occupational therapy is primarily focused on hand dexterity, not on shoulder or elbow active movement. This is to confirm the hypothesis that the current reaching-robotic intervention, primarily focused on shoulder and elbow function, improves the ability to flex and extend the shoulder and elbow joints.

### Outcome measures

#### Clinical measures

Outcome measures were assessed before and after 2 weeks of intervention. A trained and experienced therapist, who had no connection with the trial, evaluated all clinical measures. The upper extremity component of the Fugl-Meyer Assessment (UE-FMA) indicates the extent of motor control development [59], with a score consisting of two parts: a wrist–hand score (range 0–24) and a shoulder–elbow score including coordination (range 0–42). The maximum UE-FMA score (66) signifies optimal recovery.

The Action Research Arm Test (ARAT) is an assessment to evaluate arm motor function [60]. It includes 19 items divided into 4 subscales: grasp, grip, pinch, and gross movement. Each item is scored from 0 to 3, with zero indicating no movement and 3 indicating normal movement. The maximum score (57) indicates optimal performance.

The modified Ashworth Scale (MAS) is used to assess the muscle tone [61] in the biceps brachii, wrist flexor, and flexor digitorum muscles. For data analysis, the MAS scores (0, 1, 1+, 2, 3, and 4) were assigned numerical values designated as “computed MAS scores” (0, 1, 2, 3, 4, and 5, respectively) [62].

#### Kinematic analysis

Kinematic analysis was carried out to evaluate the quality of the reaching movement. Two video cameras were set on the upper frame of the reaching robot system, on the upper right and upper left in front of the patients. Reaching movements with the paretic upper limbs were recorded with a three-dimensional motion analysis system with KinemaTracer (KISSEI COMTEC Co., Ltd. Matsumoto, Japan) before and after the 2-week intervention. For assessing individual and intra-joint movements, reflective markers were placed on the acromion process, the lateral epicondyle, and the radial styloid process representing the shoulder, elbow, and wrist movements, respectively. This protocol is similar to those of previous studies (only 3 markers on the limb) [63–65], even though the definition of the joint angular is different.

The reaching movement was defined as a continuous movement from the start button to the target button (forward path), and from the target button back to the start button (backward path). The elbow extension during the reaching movement was defined as the angle between the vector of the acromion process and the lateral epicondyle, and the vector of the lateral epicondyle and the radial styloid process. The movements of shoulder and wrist were postulated as the movement of the acromion process and the radial styloid process, respectively.

**Fig. 6:**
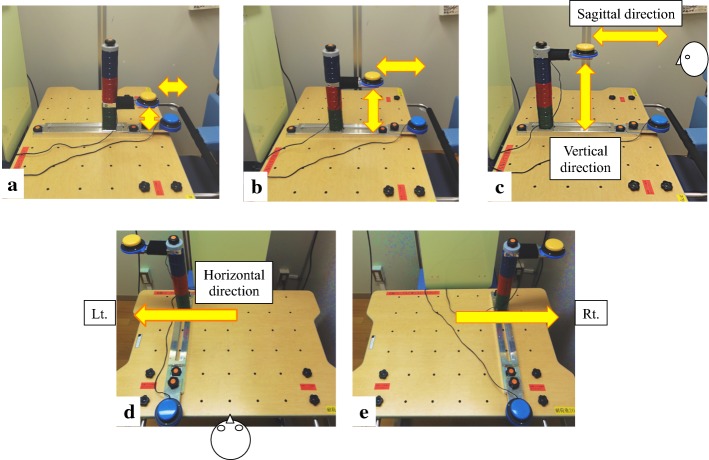
Five different positions for the start button (blue) and target button (yellow) (**a**–**e**) during each of 5 tasks for the kinematic analysis before and after 2 weeks intervention. The target button is set 10 cm (**a**), 20 cm (**b**), or 30 cm (**c**) in the sagittal and vertical directions, seen from the left side. In the ipsilateral and contralateral conditions (**d**) and (**e**), the target button is set 30 cm away in the sagittal, vertical, and horizontal directions. Ipsilateral here means the same side as the affected limb in the patient

For the assessment of kinematic analysis before and after intervention, each patient was required to perform the task movement—reaching movement between the start button and the target button—about five times without using the beneficial components of this robot (support of arm weight, NMES, and FVS). The target button was placed in five different positions: 10 cm, 20 cm, and 30 cm away (3 positions) from the start button in the sagittal and vertical directions, and 30 cm away in the sagittal, vertical, and horizontal directions (2 positions)—namely, in the ipsilateral and contralateral workspace, respectively (Fig. 6). The measurement was terminated if the patient could not touch the target button even once. As a reference, the same task was conducted with the non-paretic upper extremity once during the intervention period.

Kinematic outcome variables during reaching movement included time of reaching movement, active range of motion (AROM) in terms of elbow angle (degrees), and trajectory lengths traced by wrist and shoulder. The AROM of the elbow was calculated as the difference between the maximum and minimum angular degrees. Shapes of trajectories for the shoulder (acromion process) and the wrist (radial styloid process) were also described. The trajectory is described as a figure seen from the lateral side, in coordinates with the vertical axis as height and the horizontal axis as forward length. The shape of the trajectory is presented as a standardized thick line and area including one standard deviation (SD), which was calculated from 4 to 6 consecutive reaching movements in all of the five conditions (the number of consecutive reaching movements differed by patient). The trajectory length was defined as the average traveling length of the wrist or the shoulder during one round trip between the start button and the target button. The trajectory length was applied to indicate smoothness of movement; the shapes of the trajectories were used to compare change of movement patterns. Forward displacement of the shoulder was measured as the distance along the horizontal axis in the Lissajous figure. Trajectory length for the shoulder and forward displacement of the shoulder can be considered as compensation via the trunk to supplement inadequate movement of the upper limb; alternatively, it might reflect a change of movement pattern as a strategy to achieve the reaching movement.

### Statistical analysis

All variables are summarized as mean and SD or SE. The UE-FMA, ARAT, and MAS scores were assessed before and immediately after 2 weeks intervention. In the same way, AROM of elbow flexion angle (degrees) and trajectory lengths with wrist and shoulder joints were assessed using kinematic analysis. All of the clinical and kinematic data were analyzed with the Wilcoxon signed-rank test to compare pre-treatment data with post-treatment data. Statistical analysis was performed with the Statistical Package for the Social Sciences (SPSS; SPSS Inc. Chicago, IL, version 18.0 for Windows), and a *p* value of < 0.05 was considered to indicate statistical significance. Effect size (ES) was calculated as *r* = *Z*/√ *N*, using the *Z* score.

## Data Availability

All data generated or analyzed during this study are included in this published article.

## Abbreviations

UE-FMA: Fugl-Meyer Assessment
ARAT: Action Research Arm Test
MAS: Modified Ashworth Scale
UMIN-CTR: University hospital Medical Information Network Clinical Trial Registry
NMES: Neuromuscular electrical stimulation
FVS: Functional vibratory stimulation
BRS: Brunnstrom recovery stage
AROM: Active range of motion
CI: Cerebral infarction
EMG: Electromyography
